# Radiographic and clinical outcomes of muenster and sugar tong splints for distal radius fractures: a comparative study

**DOI:** 10.1186/s12891-024-07362-9

**Published:** 2024-04-03

**Authors:** Young-Hoon Jo, Myoung Keun Lee, Young Seok Lee, Wan-Sun Choi, Joo-Hak Kim, Jiwhan Kim, Chang-Hun Lee

**Affiliations:** 1 Department of Orthopaedic Surgery, Hanyang University Guri Hospital, Guri, Republic of Korea; 2 Department of Orthopaedic Surgery, College of Medicine, Hanyang University, 222, Wangsimni-ro, Seongdong-gu, Seoul, 04763 Republic of Korea; 3 Department of Orthopaedic Surgery, College of Medicine, Ajou University, Suwon, Republic of Korea; 4 Department of Orthopaedic Surgery, Myongji Hospital, Goyang, Republic of Korea

**Keywords:** Distal radius fracture, Conservative management, Sugar tong splint, Muenster splint, Radiologic outcome

## Abstract

**Background:**

Non-operative management is typically indicated for extra-articular distal radius fractures. Conservative treatments such as Sugar tong splints (STs) and Muenster splints (MUs) are commonly used. However, there is limited research and outcome data comparing the two splint types. Therefore, this study aimed to investigate and compare the radiographic and clinical outcomes of treatment using STs and MUs.

**Methods:**

In this retrospective comparative study, we aimed to evaluate and compare the radiographic and clinical outcomes of STs and MUs for the treatment of distal radius fractures. The study included 64 patients who underwent closed reduction (CR) in the emergency room and were treated with either STs or MUs splints (STs group: *n* = 38, MUs group: *n* = 26). Initial X-rays, post-CR X-rays, and last outpatient follow-up X-rays were evaluated. Radial height (RH), ulnar variance (UV), radial inclination (RI), and volar tilt (VT) were measured by a blinded investigator. The Quick DASH form was applied to measure patients’ satisfaction after treatments.

**Results:**

There were no significant differences in baseline characteristics, initial radiographic measurements, or radiographic measurements immediately after CR between the two groups. However, the overall radiological values deteriorated to some degree in both groups compared to the post-CR images. Furthermore, using a paired test, the STs group showed significant differences in RH and RI, and the MUs group showed significant differences in RH and UV between the last follow-up and post-CR images.

**Conclusions:**

The study concluded that there was no difference in clinical outcomes between the two splint types. However, both STs and MUs groups showed reduced radiographic parameters, and the MUs group showed a significant reduction of RH and UV in the treatment of distal radius fractures.

**Level of evidence:**

Level IV; Retrospective Comparison; Treatment Study.

## Introduction

Distal radius fractures are one of the most common types of fractures encountered in clinical practice. These fractures account for 1.5% of all emergency department visits in the United States [1]. Treatment options for distal radius fractures can be divided into surgical and non-surgical treatments. Non-surgical treatment is typically required for extra-articular fractures (AO classification 2R3A), whereas surgery is performed in approximately half of the complete intra-articular fractures (AO classification 2R3C) cases [2]. Conservative treatment has been performed with several types of splints or cast including radial gutter splint, sugar tong splints (STs), volar dorsal splint, short arm casts, and long arm cast [3–5]. The STs is a widely used stabilization method for the conservative treatment of DRF; however, patient discomfort and poor outcomes are commonly associated with this treatment due to the restriction of elbow joint movement [6, 7].

To overcome these limitations of STs, we have attempted to apply Muenster splints (MUs) to stable distal radius fractures since 2020. Muenster splint which does not cover the elbow joint and allows flexion-extension motion of the elbow, is different from the sugar tong splint. Given that a joint excursion from 130° of elbow flexion to 30° of elbow extension and 50° of supination to 50° of pronation is required for daily life function [8, 9], it is possible that the MUs may offer more range of motion to the patient because it allows elbow motion. However, there is a concern that it may disrupt the secure holding power at the fracture site.

To date, no study has compared the radiological and clinical results of MUs and STs. Therefore, we aimed to investigate the efficacy of both splints in treating stable distal radius fractures. By comparing the radiological and clinical outcomes of these two treatments, we hope to determine the optimal management strategy for these common fractures. This study hypothesizes that for stable distal radius fractures, using MUs, which allow elbow motion to improve daily activities, will not yield significantly different radiographic and clinical outcomes compared to using STs.

## Materials and methods

### Study population

This retrospective comparative study was conducted by reviewing patient data from our hospital’s electronic medical records between January 2008 and May 2022. We screened patients by searching for prescription codes corresponding to the STs and MUs.

The study included patients who presented to the emergency room with acute stable distal radius fractures and were managed with closed reduction and splint immobilization. Stable distal radius fractures were defined by radial shortening of less than 4 mm, volar tilt (VT) of less than 10°, articular comminution of less than 50%, and intra-articular step-off of less than 2 mm.

We excluded patients with unstable distal radius fractures, pediatric fractures, concomitant forearm fractures, short follow-up periods (less than 2 months after trauma), and those with incomplete or unreadable medical records. Patients managed with splinting devices other than the STs and MTs, such as a short-arm splint, short-arm cast, or long-arm cast, were also excluded. Finally, 64 patients were included in the study. Among these, 38 were assigned to the STs group and 26 to the MUs group.

This study was approved by the institutional review board of the hospital (HYUH-2022-05-018), which waived informed consent because the study involves no more than minimal risk to the subjects. However, we obtained verbal informed consent from all patients through telephone interviews.

### Management

All patients included in this study received closed reduction in the emergency room during their first visit. According to our data, patients treated before 2020 were maintained with STs (STs group), while those treated after 2020 had their splints changed to MUs (MUs group). Before 2020, all patients with a distal radius fracture visiting our emergency room were applied with STs after closed reduction. However, since 2020, MUs have been applied to all patients to allow elbow motion. Patients were not allowed to remove the splints themselves, and the total immobilization period was approximately 4 weeks. Subsequently, active range of motion exercises of the wrist were initiated immediately without additional brace or removable splint. Patients were permitted to use their hands in daily activities but were not allowed to lift heavy weights or perform vigorous activities.

The follow-up periods varied individually, and all included patients were followed up for at least 2 months. The mean follow-up period was approximately 11 weeks after injury. At every follow-up, X-ray evaluations were conducted, and we used the initial image obtained in the emergency room, the image obtained after closed reduction, and the image obtained at the last follow-up.

### Splint protocol

**Fig. 1:**
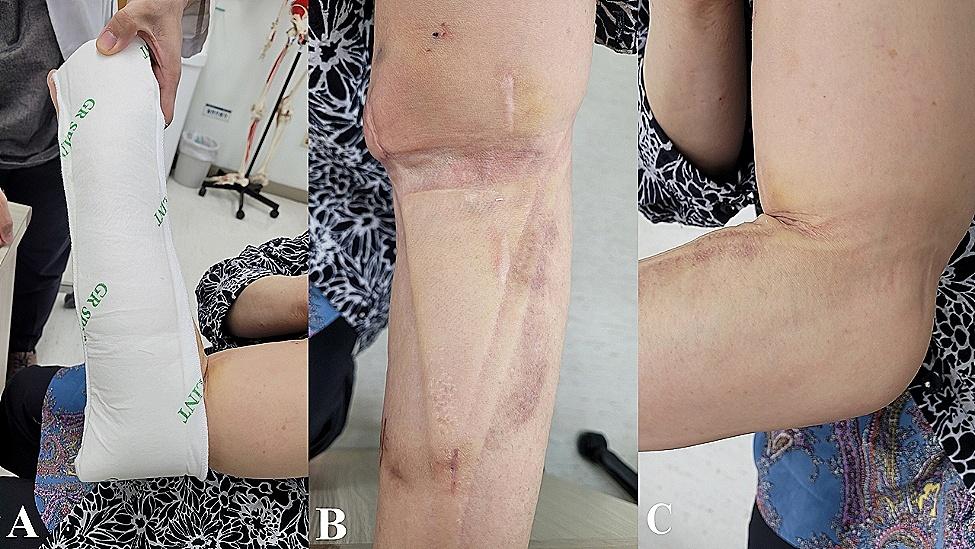
**A**: Patient in a sugar tong splint (elastic bandage is not applied); **B, C**: elbow joint is restricted by the splint

**Fig. 2:**
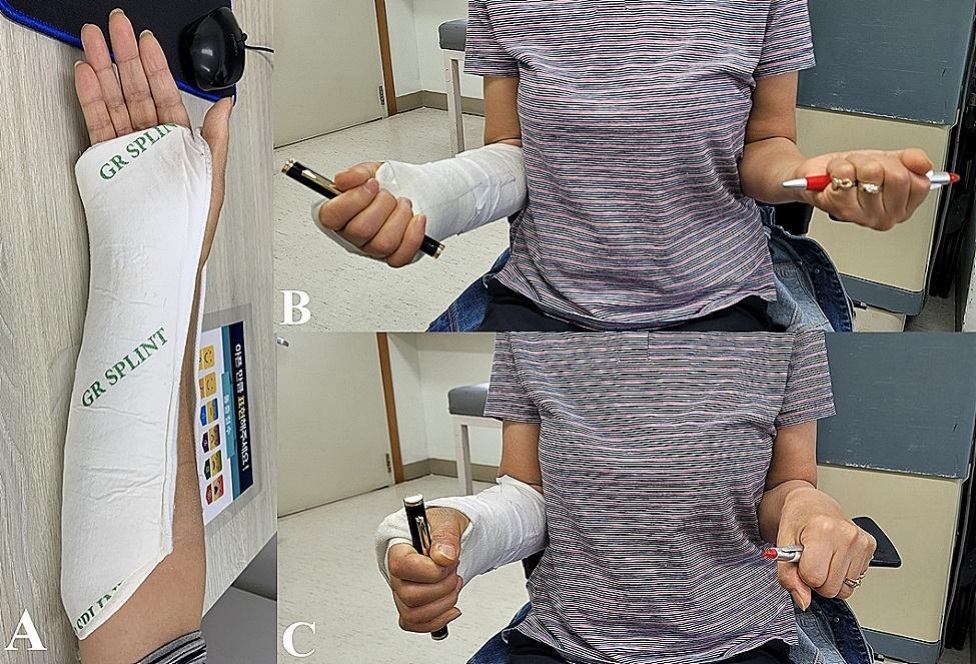
**A**: Patient in a Muenster splint (elastic bandage is not applied); **B, C**: Patients showing supination and pronation movement in the splint

The splint used in this study was moulded from non-woven fabric splint (DUK-In N-Splint®, Gyeonggi-do, Korea), with a size of 3” × 35” (7.5 cm x 89 cm). The STs covered the dorsal metacarpophalangeal joints in neutral rotation of the wrist, extending along the forearm to the humerus over the lateral epicondyle, above the olecranon process, over the medial epicondyle, to the volar aspect of the forearm, and finally to the distal palmar crease with the thenar eminence cleared (Fig. 1). On the other hand, the MUs had splinting material extending from the distal palmar crease to the proximal 1/5 of the forearm, with the wrist in neutral rotation (Fig. 2).

### Patient data and radiologic evaluation

**Fig. 3:**
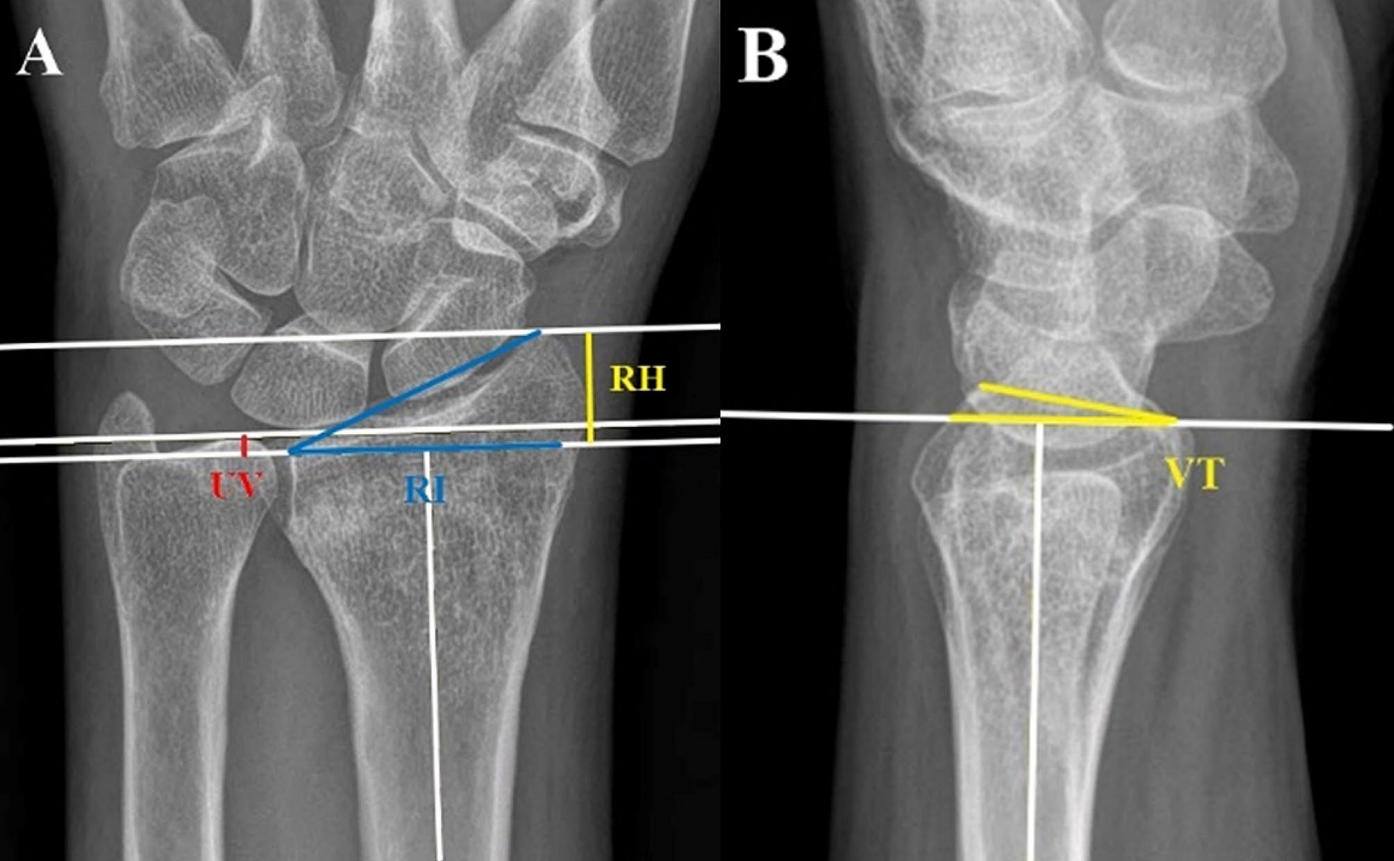
Radiological parameters **A**: Radial height (RH) is the distance between two parallel lines drawn perpendicular to the long axis of the radial shaft, one from the tip of the radial styloid and the other from the ulnar corner of the lunate fossa. Radial inclination (RI) is the angle between one line connecting the radial styloid tip and the ulnar aspect of the distal radius and a second line perpendicular to the longitudinal axis of the radius. Ulnar variance (UV) refers to the relative lengths of the distal articular surfaces of the radius and ulna. **B**: Volar tilt (VT) is the angle between a line along the distal radial articular surface and the line perpendicular to the longitudinal axis of the radius

Baseline data including age, sex, alcohol consumption, smoking status, diabetes mellitus, and chronic kidney disease were extracted to evaluate the characteristics of the study population. The initial wrist anteroposterior and lateral views obtained in the emergency room were used to determine AO classifications 2R3A, 2R3B, and 2R3C. Radiological images were obtained at each visit to measure radial height (RH), ulnar variance (UV), radial inclination (RI), and VT (Fig. 3) [6]. Non-union was indicated by the presence of a radiolucent line and symptoms such as pain or tenderness were recorded on the final follow-up sheet. A web-based picture archiving and communication system (PACS) was used, and two blinded researchers independently evaluated the X-rays.

### Clinical assessments

A questionnaire form was used to assess patient satisfaction (Quick DASH) [10]. A phone call survey was conducted approximately 6 months after the last follow-up.

### Sample size

The primary outcome of the current study was the mean VT at final follow up. In a previous study, significant differences in VT were observed at the final follow-up among patients with distal radius fractures undergoing conservative treatment with short arm casts compared to those with long arm casts [11]. While this study utilized splints for treatment, the key distinction between the STs and MUs lies in restricting the motion of the elbow. Therefore, anticipating results similar to the previous study, we calculated the sample size based accordingly. In previous study, the mean final VT and its standard deviation (SD) for the short arm cast group were − 3.6° and 5.6°, respectively, while for the long arm cast group, they were 2.3° and 6.2°, respectively [11]. In relation to the statistical model of independent Student’s t-test, the standardized effect size is the difference between means, divided by pooled SD of two groups [12, 13]. The standardized effect size calculated based on the above-mentioned mean VT and their SD described on the literature was 1.00; (2.3 – (–3.6))/5.91 = 1.00, 5.91 is a pooled SD of two groups. With a significance level of 0.05, power of 90%, and standardized effect size of 1.00, the required sample size was determined to be 23 cases in each group for comparison between the two groups in mean final VT. Therefore, our sample size of 38 cases in STs Group and 26 cases in MUs satisfied the required sample size.

### Statistics

The data collected were entered into Excel spreadsheets, and statistical analyses were performed using the Statistical Package for Social Sciences (SPSS) version 20 (Armonk, NY, USA). Descriptive statistics were used to summarize the continuous variables among groups as means ± standard deviation, while categorical data were presented as ratios. The Mann–Whitney U test was used to analyse continuous non-parametric variables, while Student’s independent t-tests were used to analyse continuous variables that conformed to a normal distribution. The Kolmogorov-Smirnov test and Shapiro-Wilk test were used to evaluate normal distribution of the data. The chi-squared test was used to analyse categorical variables. Since our study focused on the efficiency of maintaining reduced fragments, we compared the radiologic status of the last follow-up with those of immediate post-reduction status. To analyse repeated measures statistics for the same individual, we used a paired t-test for parametric variables that conformed to a normal distribution and the Wilcoxon signed-rank test for non-parametric variables. A *p*-value of less than 0.05 was considered statistically significant.

## Results

The study population consisted of 64 patients with an average age of 58.2 years (range: 21–85 years). The mean follow-up period was 74.7 days. Women were predominant in both groups (65.8% in STs and 76.9% in MUs group). AO 23 A type fractures had the highest percentage in both groups (52.6% in STs and 57.7% in MUs group). Patients maintained the splints for slightly less than 4 weeks (3.8 weeks in STs and 3.7 weeks in MUs group). The first follow-up after ER discharge was approximately 4 days. The follow-up times for STs and MUs groups were 70.8 days and 83.5 days, respectively. No significant differences were observed for these variables (all *P* > 0.05) (Table 1).

**Table 1:** Baseline characteristics

	Sugar tong splint (*N* = 38)	Muenster splint (*N* = 26)	***P***-value
Age (years)	58.1 ± 24.3	58.3 ± 14.7	0.635
Sex (M:F)	13:25	6:20	0.411
AO classification (A:B:C)	20:9:9	15:3:8	0.461
Immobilized period (weeks)	3.8 ± 1.6	3.7 ± 0.9	0.802
Smoker*† (No:Yes)	14:4	9:0	0.268
Alcohol*† (No:Yes)	15:3	8:1	0.593
DM*† (No:Yes)	13:5	8:1	0.628
CKD*† (No:Yes)	16:2	8:1	1.000.
Period to 1st OPD (days)	4.0 ± 3.0	3.1 ± 2.3	0.455
Period to Last f/u (days)	70.8 ± 35.9	83.5 ± 34.1	0.760

Mann–Whitney test for continuous nonparametric variables

Chi-squared test for nominal variables

*Fisher’s exact test

†Loss of total number due to absence of records

Continuous variables are presented as mean ± standard deviation

Radiological parameters were measured on initial X-rays. RH was 10.8 mm for STs and 10.7 mm for MUs groups. UV was 0.8 mm for STs and 1.3 mm for MUs groups. RI was 22.5° for STs and 22.5° for MUs groups. VT was 5.3° for STs and 3.1° for MUs groups. None of the initial measurements showed significant differences (all *P* > 0.05) (Table 2). Intra- and interobserver reliabilities of initial measurements were assessed using intraclass correlation coefficient (ICC). All Cronbach’s alpha values were above 0.930, and all *P*-values were < 0.001 (Table 3).

**Table 2:** Radiologic measurements at three different stages: initial presentation, immediately after reduction, and final follow-up

	Sugar tong splint (*N* = 38)	Muenster splint (*N* = 26)	d*	***P***-value
**Initial Presentation**				
Radial height (mm)	10.8 ± 2.1	10.7 ± 1.7	0.052	0.770
Ulnar variance (mm)	0.8 ± 1.9	1.3 ± 1.5	0.292	0.275
Radial inclination (°)	22.5 ± 4.8	22.5 ± 3.1	0	0.763
Volar tilt (°)	5.3 ± 10.3	3.1 ± 9.5	0.222	0.397
**Immediately After Reduction**				
Radial height (mm)	12.1 ± 2.9	11.3 ± 2.0	0.321	0.347
Ulnar variance (mm)	0.8 ± 1.3	1.0 ± 1.7	0.132	0.975
Radial inclination (°)	23.4 ± 4.6	22.5 ± 3.9	0.211	0.165
Volar tilt (°)	7.0 ± 8.6	3.8 ± 8.8	0.368	0.112
**Final Follow Up**				
Radial height (mm)	11.1 ± 2.6	10.3 ± 1.8	0.358	0.309
Ulnar variance (mm)	1.0 ± 1.9	1.6 ± 1.8	0.324	0.397
Radial inclination (°)	22.1 ± 4.9	21.8 ± 3.9	0.068	0.654
Volar tilt (°)	5.9 ± 10.1	2.3 ± 8.1	0.393	0.184

Independent t-test for parametric variables

*d means Cohen’s d. Cohen’s d is an effect size used to indicate the standardised difference between two means

Variables are presented as mean ± standard deviation

**Table 3:** Intraclass correlation coefficient (ICC) evaluation for intra- and inter-observer reliability

	Intra-observer reliability	***P***-value	Inter-observer reliability	***P***-value
Radial height	0.962	**< 0.001**	0.942	**< 0.001**
Ulnar variance	0.986	**< 0.001**	0.937	**< 0.001**
Radial inclination	0.970	**< 0.001**	0.979	**< 0.001**
Volar tilt	0.998	**< 0.001**	0.983	**< 0.001**

Mann–Whitney U test was used

Statistically significant values are in bold

All patients underwent post-reduction X-rays. RH was 12.1 mm for STs and 11.3 mm for MUs groups. UV was 0.8 mm for STs and 1.0 mm for MUs groups. RI was 23.4° for STs and 22.5° for MUs groups. VT was 7.0° for STs and 3.8° for MUs groups. No significant differences were observed in these measurements (Table 2).

**Fig. 4:**
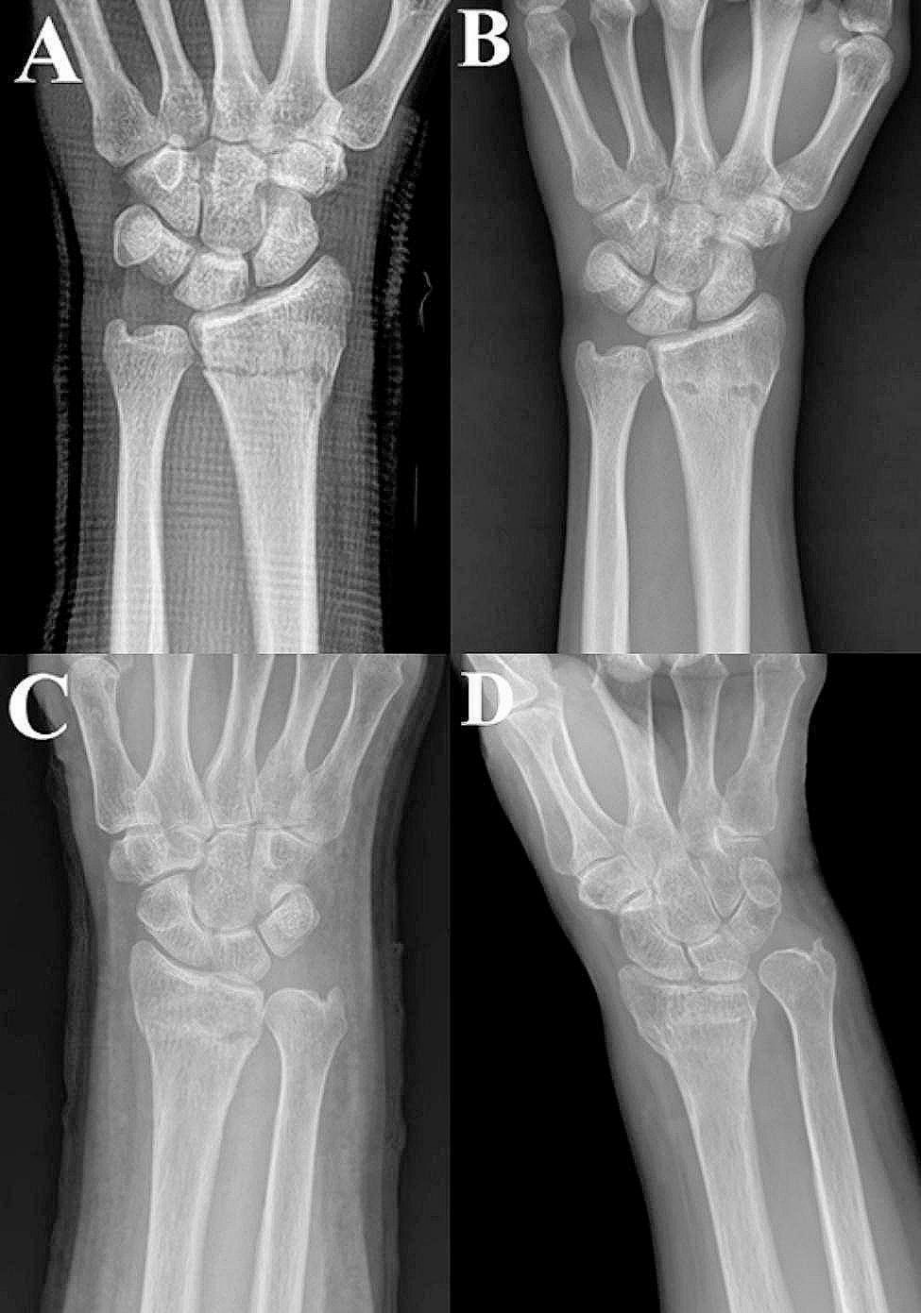
Muenster splint with a good radiological result (**A**: immediate after closed reduction, **B**: at final follow-up) and with a poor result (**C**: immediate after closed reduction, **D**: at final follow-up)

Each patient was lost to follow-up at different time points. In the last follow-up X-rays, the RH was 11.1 mm for STs and 10.3 mm for MUs group. UV was 1.0 for STs and 1.6 for MUs groups. RI was 22.1 for STs and 21.8 for MUs groups. VT was 5.9 for STs and 2.3 for MUs groups (Fig. 4). Comparisons of the means for each item were not significant (Table 2). None of the patients in our cohort developed nonunion.

Additional statistical analyses focused on repeated measures in individual patients (Table 4). In the STs group, RH and RI were significantly increased, while RH and UV were significantly increased in the MUs group. Although some *p*-values were not below 0.05, all measured parameters worsened at the final follow-up. For clinical outcome measurements, Quick DASH was conducted. The average Quick DASH scores were 0.6 for the STs and 1.3 for the MUs groups. There was no significant difference in Quick DASH scores between the two groups (*P* = 0.531).

**Table 4:** Effect of each splint in an individual (individually paired exam)

	Sugar tong splint	d*	***P***-value	Muenster splint	d*	***P***-value
Radial height (mm)	12.1 ± 2.9 → 11.1 ± 2.6	0.362	**0.030**	11.3 ± 2.0 → 10.3 ± 1.8	0.524	< **0.001**†
Ulnar variance (mm)	0.8 ± 1.3 → 1.0 ± 1.9	0.119	0.104	1.0 ± 1.7 → 1.6 ± 1.8	0.342	**0.005**†
Radial inclination (°)	23.4 ± 4.6 → 22.1 ± 4.9	0.273	**0.014**	22.5 ± 3.9 → 21.8 ± 3.9	0.179	0.101†
Volar tilt (°)	7.0 ± 8.6 → 5.9 ± 10.1	0.117	0.171†	3.8 ± 8.8 → 2.3 ± 8.1	0.177	0.334†

Because of non-normal distribution, the Wilcoxon signed-rank test was used

†Paired t-test was performed as it was matched to a normal distribution

*d means Cohen’s d. Cohen’s d is an effect size used to indicate the standardised difference between two means

Variables are presented as mean ± standard deviation. Statistically significant values are in bold

## Discussion

Distal radius fractures are among the most common fractures encountered in emergency rooms. One study reported that these fractures accounted for up to 18% of all fractures in individuals over 65 years of age [14]. In South Korea, an average of about 130,000 distal radius fractures occur annually, with a higher incidence among women aged 50 and older [15]. Non-surgical management of distal radius fractures in the older population has been debated for several decades to avoid the morbidity and costs associated with surgery. Several studies have reported non-inferiority of non-surgical treatment compared to surgical fixation [16, 17].

Our study focused on successful non-surgical treatment of these fractures, aiming to determine the optimal immobilization technique. Several studies have reported no significant difference between below-elbow casts and above-elbow casts in the management of extra-articular distal radius fractures [4–6]. An Italian randomized controlled trial reported no significant differences between the treatment groups, despite not involving a direct comparison of parameters [5]. The study used noninferiority thresholds, which indicated stability if the parameters did not exceed certain values, such as an RH of 2 mm, RI of 3°, and VT of 3°. While this approach simplifies the comparison by setting specific points, it raises concerns about the reliability of the threshold and potential biases due to missed subtle differences.

In a prospective randomized trial, Caruso et al. [6] compared the outcomes of below-elbow and above-elbow casts and found no significant differences. However, their study was limited to specific types of extra-articular distal radius fractures (AO 2R3A2.2), which reduces the applicability of their findings. A recent study comparing volar-dorsal splints and STs in the conservative treatment of distal radius fractures used flawed statistical methods [18]. The study’s flaw was in only conducting a simple comparison at each time point following closed reduction, without comparing changes over time within each group.

Our findings revealed no significant differences in radiologic parameters at any time point, including post-reduction and the final follow-up. However, when examining individual-paired comparison analysis, a trend toward worsening overall radiology was observed. RH deterioration was significant in both splints, and RI worsened in the STs group, while UV became more noticeable in the MUs group.

The MUs does not limit the flexion-extension motion of the elbow, and since muscles related to elbow movement, such as the brachialis and triceps brachii, primarily originate from the humerus and insert into the ulna and the biceps brachii, brachioradialis, and pronator teres originate from the humerus and insert into the radius, increased elbow motion may directly contribute to the radius and ulna translation when using the MUs. A comparative study suggested that STs more effectively restricted pronation than MUs [19]. As the forearm pronates, the radius shifts proximally rather than merely rotating in the ulnar sigmoid notch [20]. STs create a “closed box” configuration by covering the metacarpals and humeral condyles, while the MUs forms a “proximally open box” configuration by leaving the humeral condyles uncovered. This difference may result in positive ulnar variance in patients treated wiht MUs.

Previous stuides on the treatment of distal radius fractures have suggested that poor radiologic measurements often indicate unfavorable outcomes [21, 22]. However, these negative clinical outcomes typically occur with radial shortening > 5 mm or dorsal tilt more than 5° [22, 23]. Such ranges were not significant in our study findings. Recent studies have proposed that radiologic measurements do not always correlate with clinical outcomes [24–26]. Our radiologic results align with these studies, and we observed good clinical outcomes with no significant differences between the two splint groups. Interestingly, Grewal et al. [27] found a direct correlation between radiological malunion and poor outcomes in patients under 65 years, while no significant correlation was observed in patients over 65 years. Another study also reported a weak correlation between radiological and functional outcomes in older populations [28]. Brogren et al. [28] attributed this phenomenon to the lower demand in older patient groups. As a significant portion of our study participants are elderly, this explanation could also be relevant to our study results.

Allowing elbow joints to move freely can be considered beneficial for patients’ daily lives. A previous study demonstrated that short-arm casts resulted in better Mayo elbow scores than long-arm casts [6]. The use of MUs may lead to the possibility of unfavorable radiologic outcomes, but the clinical results have proven to be favorable. Our study could not conclusively show that the MUs group had more elbow motion in the measured degree, as we did not measure the elbow’s range of motion. MUs offer the advantage of increased elbow mobility, as shown in Fig. 2, which reduces inconvenience in daily activities during the splint immobilization period. Clinicians should be able to discuss the treatment process and outcomes with patients and choose an appropriate option based on individual need.

Our study presents several strengths. Firstly, we aimed to accurately illustrate the differences by comparing the raw numbers. Secondly, we conducted repeated measurements for each individual and employed statistical methods focused on the amount of change for each participant (paired T-test and Wilcoxon signed-rank test). Thirdly, we included all radius fractures deemed as stable fractures in our study without limiting the fracture type (AO classification) or age of the patients to old age.

However, our study has some limitations, such as the retrospective data collection and potential selection bias due to the exclusion of many consecutive data points. Although the difference was statistically significant, the average difference of approximately 1 mm or 1° may introduce errors in the measurement technique. Nonetheless, two researchers independently conducted measurements to ensure the reliability of the ICC values. Another limitation is the lack of objective clinical measurements, such as grip strength [29]. Quick DASH is a subjective patient evaluation, not an objective patient evaluation. Our study could not include grip strength measurements, as they were not recorded before 2020.

## Conclusions

In conclusion, our study demonstrated no significant radiological differences between the STs and MUs groups at any time point. However, we observed a tendency for radiological parameters to worsen over time in both groups. Comparing individual patients, we found that MUs were associated with worse radiological outcomes, particularly in terms of UV. Although worse radiological parameters might be related to the use of MUs, there were no significant differences in functional outcomes. Considering the convenience provided by the mobile elbow joint, we recommend the utilization of MUs in the treatment of stable distal radius fractures.

## Data Availability

The datasets during and/or analyzed during the current study are available from the corresponding author on reasonable request.

## Abbreviations

STs: Sugar tong splints
MUs: Muenster splints
VT: volar tilt
RH: radial height
UV: ulnar variance
RI: radial inclincation
SD: standard deviation
ICC: intraclass correlation coefficient
